# 

**Published:** 1892-05

**Authors:** 


ESTABLISHED 1855. SUBSCRIPTION, $1.00

LIBRARY
Indexed
S. G. O.

### ATLANTA MEDICAL AND SURGICAL JOURNAL.

OLD SERIES, VOL. XXVI. }

NEW SERIES, VOL. IX. } ATLANTA, GA., MAY, 1892. NO. 3.

LUTHER B. GRANDY, M. D., MANAGING EDITOR.

M. B. HUTCHINS, M. D., BUSINESS MANAGER.

